# Discrepancies between the drivers of alpha and beta plant diversity in arable field margins

**DOI:** 10.1098/rspb.2022.2179

**Published:** 2023-02-08

**Authors:** Sébastien Boinot, Audrey Alignier

**Affiliations:** ^1^ UMR 0980 BAGAP, INRAE - Institut Agro - ESA, 65 rue de St Brieuc CS 84215, 35042 Rennes, France; ^2^ LTSER 'Zone Atelier Armorique', 35042 Rennes, France

**Keywords:** agricultural intensification, biotic homogenization, functional diversity, hedgerow, long-term management, weed

## Abstract

Field margins are major habitats for biodiversity conservation and ecosystem functioning in agricultural landscapes, but biotic homogenization of plant communities threatens their ecological and agronomic functions. Our objective is to determine the drivers of plant diversity in field margins for conservation and restoration purposes. To do so, we assessed the effects of field margin structure and long-term management over 20 years (1995–2015) on the taxonomic and functional α- and β-diversity, and the functional composition of herbaceous plant communities. In 2015, we surveyed 302 field margins in bocage landscapes of Brittany, northwestern France. Results were very similar between taxonomic and functional diversity but revealed important discrepancies between the drivers of α- and β-diversity. Deep ditches, mowing and grazing increased α-diversity but did not affect β-diversity. Denser hedgerows had lower α-diversity than other field margins but strongly contributed to β-diversity by harbouring more unique sets of species or life strategies. Long-term herbicide spraying in field margins and cropping intensity in adjacent habitats did not affect α-diversity, but had more complex effects on β-diversity and selected for common weeds. All in all, preservation of dense hedgerows, abandonment of herbicide spraying, and protection against agrochemical drifts are key measures to prevent the establishment of common weeds and biotic homogenization of herbaceous plant communities in field margins. Above all, our study shows how important it is to go beyond α-diversity to make robust conservation and restoration decisions.

## 1. Introduction

Field margins are semi-natural habitats that border arable fields. These habitats are very important for biodiversity conservation and ecosystem service provision in agricultural landscapes, as they are generally more stable and less affected by agricultural disturbances than cropland [1,2]. As a result, field margins allow the survival, establishment and dispersal of many plant and animal species that would otherwise disappear from intensive production areas [3]. Beyond its intrinsic value, the preservation of plant diversity in field margins is a prerequisite for maintaining a variety of ecosystem services in space and time [4]. Indeed, a diversity of plant species provides different trophic and habitat resources for wildlife, including natural enemies of crop pests, pollinators and detritivores [5]. In addition, plant species contribute in different ways to nutrient and water cycling, climate regulation, pollution control and erosion protection, among other ecosystem services (e.g. [6,7]).

Most studies on plant diversity conservation in field margins have focused on taxonomic α-diversity, i.e. the total diversity of species measured *at field margin scale*. It is well-established that taxonomic α-diversity of plant communities is affected by field margin management and structure, adjacent land use, and landscape context (e.g. [8–10]). Notably, it appears that chemical-intensive agriculture is threatening plant diversity of field margins, through direct habitat destruction or indirect effects on plant communities (e.g. agrochemical drifts from cropland and eutrophication of field margins) [8,11]. To our knowledge, most studies used a ‘snapshot’ approach (over a season or year), whereas the effects of cumulative practices over decades remain largely unknown. Moreover, we do not know how changes in local α-diversity scale up to plant diversity conservation at larger spatial scales (γ-diversity). Very few studies have measured β-diversity of field margins *at landscape scale*, which is the heterogeneity of community composition between field margins in agricultural landscapes. Changes in β-diversity provide the scaling factor that allows us to predict changes in γ-diversity from measured changes in α-diversity [12]. Reduction in β-diversity indicates biotic homogenization, an increasingly recognized threat to biodiversity conservation and ecosystem functioning, resistance and resilience [13]. Importantly, previous studies reported divergent responses of α- and β-diversity across a variety of taxa and ecosystems (e.g. [14–16]), which highlights the importance of considering β-diversity when making conservation or restoration decisions. In addition, studies at larger spatial scales (i.e. landscape or beyond) more often find evidence of biodiversity extinction events, indicating that conservation efforts must be considered at these scales ([12] and references therein).

The approach of the few studies that measured β-diversity of herbaceous plant communities in field margins was to compare β-diversity levels over time. Using long-term data on plant species richness over 70 years, Staley *et al*. [17] revealed that biotic homogenization of herbaceous plant communities has indeed occurred in hedgerows. The authors also found that nitrophilous and competitive species are becoming more common and dominant, possibly owing to changes in hedgerow management and eutrophication. Conversely, other studies did not find evidence for biotic homogenization in hedgerows or herbaceous field margins [18–20]. It was hypothesized that the diversity of field margin structures and management practices were maintaining high β-diversity. However, no study has yet determined the drivers of spatial β-diversity of plant communities in field margins. It is unclear which margin structures and management practices contribute most to maintaining plant diversity across field margins (at landscape scale), and whether there are antagonisms or synergies between the conservation of α- versus β-diversity.

In this study, we revisited the data analysed by Alignier [19] to assess the effects of field margin structure (i.e. physical aspects such as tree cover and margin width) and long-term management of herbaceous vegetation over 20 years (i.e. disturbance regimes including for example herbicide spraying and mowing frequency) on both α- and β-diversity of herbaceous plant communities. We sampled a broad range of field margins simultaneously—from herbaceous margins to dense hedgerows—in order to (1) disentangle confounding factors (e.g. margin structure versus long-term management of herbaceous layer), and (2) compare the relative importance of margin types in maintaining plant diversity in the landscape. Further, we measured both taxonomic and functional diversity, which do not necessarily show similar patterns and provide complementary information regarding plant community assembly (e.g. [21]). Functional diversity should provide a more accurate estimate of ecosystem functioning, resistance and resilience given that species traits determine their ecological functions, their interaction with other species, and their response to environmental gradients [22]. By describing the functional *composition* of plant communities (i.e. the presence and relative abundance of certain trait values), we are also able to infer the mechanisms driving community assembly and the implications for biodiversity conservation and agricultural production. On the other hand, these advantages come at a price: trait choice is critical as it can strongly affect the ecological conclusions drawn from functional diversity measures [23]. We specifically addressed the following questions: (1) Which margin structures and long-term management practices contribute most to maintaining plant diversity at local scale (α-diversity) versus landscape scale (β-diversity)? (2) Which plant life strategies are selected along these environmental gradients? (3) Are there any antagonisms or synergies between the conservation of α- versus β-diversity, or taxonomic versus functional diversity? Previous studies have shown that structurally simple, herbaceous, flat and narrow field margins, as well as most disturbed ones, are more prone to harbour weedy species [24–26]. We suspected that the selection for weedy life strategies in such field margins might lead not only to local impoverishment (i.e. a decrease in taxonomic and functional α-diversity) but also to strong biotic homogenization in the landscape (i.e. a decrease in taxonomic and functional β-diversity).

## 2. Material and methods

### (a) Study site

**Figure 1.**
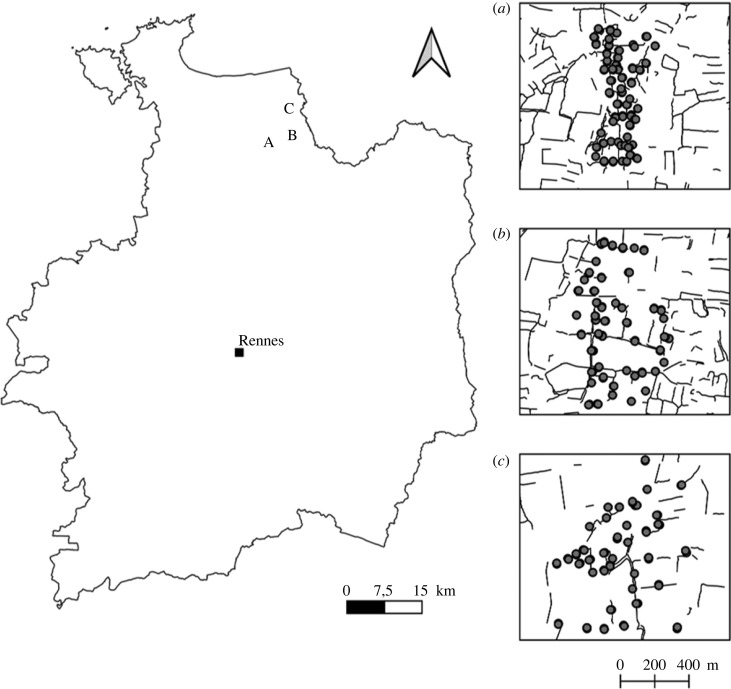
Geographical position of the study area in the department of Ille-et-Vilaine, Brittany, France. Grey points indicate the location of sampled field margins (*n* = 302) in the three contrasting landscapes (*a*, *b* and *c*). Black lines in each landscape window represent the hedgerow network, based on the national reference layer of hedgerows in metropolitan France provided by the National Institute of Geographical and Forest Information.

We conducted the study in the Long-Term Socio-Ecological Research site ‘Zone Atelier Armorique’ in Brittany, northwestern France (48° 36′ N, 1° 32′ W; figure 1). The Zone Atelier Armorique comprises three contrasting hedgerow network landscapes (A, B and C) also called ‘bocage’ (electronic supplementary material, figure S1). These three landscapes range from dense bocage with small fields in the south to more open bocage with larger fields in the north (figure 1). Ancient hedgerows are mainly composed of oak or chestnut trees generally planted on a bank surrounded by ditches. The shrub layer is rarely continuous and may even be absent. Agricultural landscapes are dominated by wheat and maize fields and temporary sown grasslands (less than 5 years) under conventional farming.

### (b) Vegetation sampling

We surveyed 302 field margins, from herbaceous to woody margins with or without shrub or tree layer (electronic supplementary material, figure S2), across the three contrasting landscapes of the Zone Atelier Armorique (106 in landscape A, 103 in landscape B, and 93 in landscape C) (figure 1). We defined a field margin as one side of the entire field boundary [19] (electronic supplementary material, figure S3). Thus, within a boundary, we distinguished two field margins for vegetation sampling, each adjoining a specific habitat (e.g. arable field, woodland, road, water). From May to July 2015, we sampled herbaceous plants in 25 m long plots (one plot per field margin) located in the middle of the field margin to avoid edge effects. We sampled the full width of field margins (varying from 0.15 to 3.8 m) to account for local heterogeneity in field margin structure (e.g. presence of banks and ditches). We visually estimated species percentage cover as a proxy for abundance using an ordinal scale from one to five [27]. We used the middle of cover classes for subsequent analyses (one: 5%, two: 14.5%, three: 37.5%, four: 62.5% and five: 87.5%).

### (c) Description of plant communities

All diversity indices were computed using species abundance data, which is recommended for studies carried out within relatively small spatial areas [28]. We measured taxonomic α-diversity using the Hill–Shannon index, which considers both the number of species within a community and their relative (proportional) abundance [29]. We measured taxonomic β-diversity among field margins using the Jaccard pairwise dissimilarity index.

Following previous studies, we described the functional diversity of plant communities using traits and ecological indicators that are related to plant establishment, reproduction and dispersal (see [11,25]). These traits and ecological indicators are also affected by disturbance regime and resource availability resulting from field margin management/structure and adjacent farming practices. More precisely, we considered the following traits: life form (annual/perennial), growth form (grass/other), specific leaf area, plant height, seed mass, pollination mode (entomogamous/other) and predominant seed dispersal modes (wind, animals, other). We also considered Ellenberg values for light, nutrients and soil moisture. We collected functional attributes and ecological indicator values in the following databases: Ecoflora [30], Baseflor [31] and LEDA [32]. We produced a functional tree from the ‘species × traits’ matrix using the ‘gawdis’ R function, which avoids a disproportional contribution of categorical traits [33]. Functional α-diversity is the total branch length of the functional tree linking all species present in each community [34]. On the other hand, functional β-diversity is the total length of branches that are unique to each community. We computed diversity indices with the package BAT [35]. Pairwise correlations and distributions of trait values are given in electronic supplementary material, figure S4.

### (d) Environmental gradients

Field margin structure was defined using seven variables measured in 2015, namely canopy height, margin width, tree cover, shrub cover, woody species richness, bank height and ditch depth. We visually estimated tree and shrub cover (%). Canopy width was also measured but removed from further analyses given its high positive correlation with canopy height (*r* = 0.82; electronic supplementary material, figure S5). On the other hand, long-term management of herbaceous plant communities in field margins was defined using four variables measured by visual observations all year round from 1995 to 2015 (electronic supplementary material, figure S6), namely the occurrence of herbicide spraying, mowing, grazing, and tillage disturbances (intentional or not). An indicator of management intensity (%) for each practice was calculated as the number of occurring events divided by the total number of visual observations for each field margin. The number of observations per field margin over 20 years ranged from 77 to 98, with a mean of 95 ± 7 observations (i.e. generally four observations per field margin per year). In addition, we calculated cropping intensity (%) in habitats adjoining field margins, measured as the number of years of cropping (i.e. excluding grasslands and fallows) divided by the total number of years. Cropping intensity not only indicates the nature of adjacent habitats (non-arable versus arable) but also provides an estimate of the accumulation of agrochemical drift events (all arable fields adjoining margins were under conventional farming). Pairwise correlations and distributions of variables are given in electronic supplementary material, figure S5.

### (e) Data analysis

First, we used generalized additive models (GAMs) to assess the effects of field margin structure and long-term management on taxonomic and functional α-diversity. GAMs are very useful to assess the nonlinearity of relationships (e.g. intermediate disturbance hypothesis). We included the coordinates (latitude and longitude) of each field margin in GAMs to account for spatial autocorrelation, using an unconstrained two-dimensional spline [25]. We included environmental gradients using splines with a limited degree of freedom (*k* = 5) to avoid overfitting (e.g. [25,36]). We standardized environmental gradients to facilitate parameter estimation and interpretation [37]. We checked the degree of collinearity between environmental gradients using a generalized pairs plot (electronic supplementary material, figure S5) and variance inflation factors (VIFs). All VIF values were lower than 3, indicating that collinearity was not an issue [38]. Note that we considered using generalized additive mixed models (GAMMs) to incorporate landscape categories (A, B and C) as a random effect on the intercept. However, Akaike information criterion (AIC) was consistently higher and adjusted *R*² lower for GAMMs, indicating that landscape category was not relevant (taxonomic α-diversity: AIC_GAMM_ = 1928 versus AIC_GAM_ = 1883, $R_{\mathrm{GAMM}}^{2}=0.24$ versus $R_{\mathrm{GAM}}^{2}=0.35$/functional α-diversity; AIC_GAMM_ = 707 versus AIC_GAM_ = 672, $R_{\mathrm{GAMM}}^{2}=0.21$ versus $R_{\mathrm{GAM}}^{2}=0.34$). We computed Gaussian GAM(M)s using the R package mgcv [39].

Second, we used generalized dissimilarity modelling (GDM) to assess the effects of field margin structure and long-term management on taxonomic and functional β-diversity. GDM is an extension of matrix regression that allows the incorporation of several explanatory variables, including geographical distance, to predict β-diversity [40]. We fitted models using the R package gdm with the default number of splines and knots [41]. We used the R function gdm.varImp to assess model significance and variable importance, using matrix permutation (*n* = 100) and backward elimination procedure. In addition, we estimated uncertainty (standard deviation) in the fitted splines using a bootstrapping approach, subsampling 80% of the dataset 100 times.

Third, we used RLQ and fourth-corner analyses to assess the effects of field margin structure and long-term management on the functional composition of plant communities [42]. Before these analyses, we removed rare species whose frequency of occurrence was lower than 2%, and we log-transformed species cover, as both very rare and very dominant species may unduly influence the results [43]. The remaining species represented 98% of the total cover of herbaceous vegetation. We also log-transformed seed mass, which was highly skewed (electronic supplementary material, figure S4). Then, we used a fourth-corner analysis to test the significance of correlations between each trait and each environmental variable. Further, we tested the correlations between RLQ axes and traits or environmental variables. For all tests, we used 49 999 random permutations of species and plots. We used the R package ade4 to perform these analyses [44]. We considered incorporating landscape categories (A, B and C) using partial RLQ analysis [45]. Again, landscape category did not seem relevant as the percentage of variance explained by the first two axes was lower for the partial RLQ analysis (89.75 versus 93.57% for the basic RLQ analysis). Finally, we classified species into non-weedy species versus common weeds, based on the French reference book *Mauvaises herbes des cultures* [46]. Then, we used the ‘s.class’ R function and Wilcoxon tests to compare the distribution of non-weedy species versus common weeds on RLQ axes.

## 3. Results

### (a) Effects of field margin structure and long-term management on α-diversity

**Figure 2.**
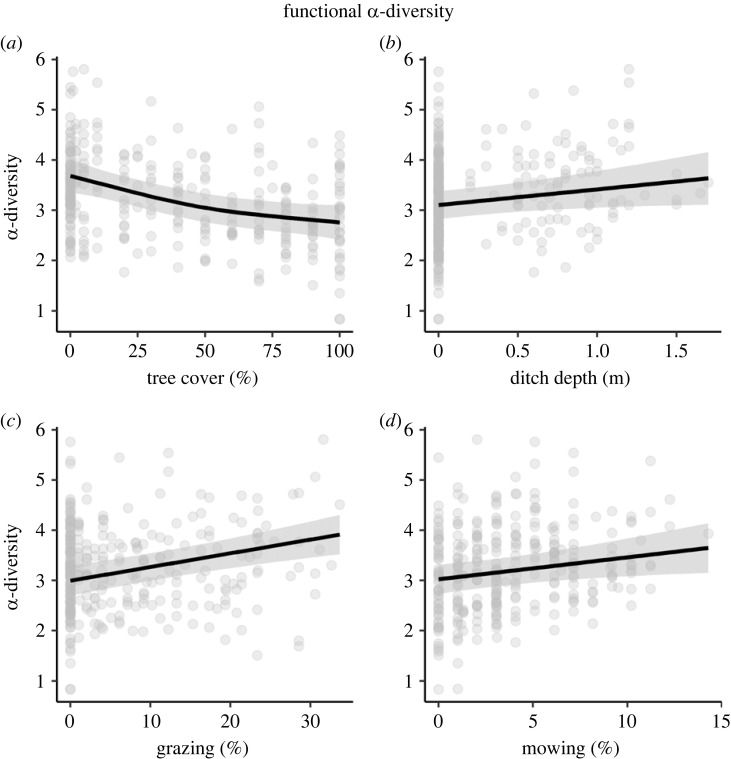
Field margin structure and long-term management both affected the functional α-diversity of herbaceous plant communities, based on the results of generalized additive models (GAMs). (*a*) Functional α-diversity decreased in an almost linear way with tree cover, whereas (*b*) ditch depth linearly increased functional α-diversity. Further, (*c*) long-term grazing and (*d*) mowing intensity both linearly increased functional α-diversity. Raw data are represented by the dots. Grey-shaded areas represent 95% confidence intervals.

Generalized additive models (GAMs) explained a significant proportion of the variation in taxonomic and functional α-diversity ($R_{\mathrm{adj}}^{2}=0.35\;\mathrm{and}\;0.34$, respectively). Results were very similar between taxonomic and functional α-diversity (electronic supplementary material, tables S1 and S2; figure 2 and electronic supplementary material, figure S7), which were strongly correlated with each other (*r* = 0.93). Thus, we focus on functional α-diversity hereafter. Regarding field margin structure, functional α-diversity decreased in an almost linear way with tree cover (figure 2*a*; electronic supplementary material, table S2), whereas ditch depth linearly increased functional α-diversity (figure 2*b*; electronic supplementary material, table S2). Functional α-diversity varied in a nonlinear way along the gradient of woody species richness (electronic supplementary material, table S2). Regarding the long-term management of herbaceous vegetation, grazing and mowing intensity linearly increased functional α-diversity (figure 2*c,d*; electronic supplementary material, table S2). Surprisingly, other covariables such as margin width or herbicide spraying and cropping intensity did not affect taxonomic and functional α-diversity (electronic supplementary material, tables S1 and S2).

### (b) Effects of field margin structure and long-term management on β-diversity

**Figure 3.**
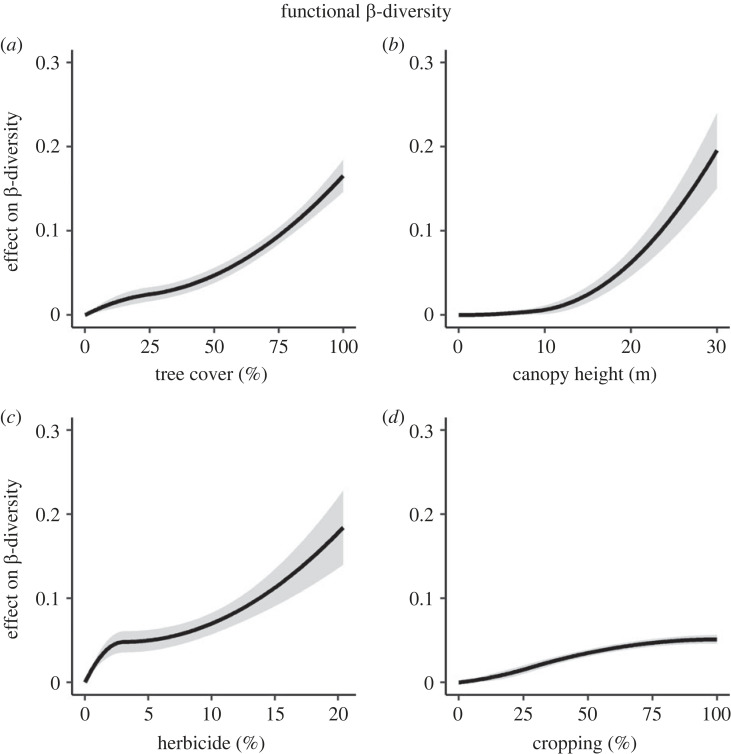
Effects of field margin structure and long-term management on functional β-diversity of plant communities in field margins, based on the results of generalized dissimilarity models (GDMs). Note that the *y*-axis does not represent β-diversity values, but the effect of each explanatory variable on β-diversity (while holding other covariates constant). Curve shapes describe the effect of each explanatory variable along its gradient (*x*-axis) on functional β-diversity. A steeper slope implies greater dissimilarity per unit change in the explanatory variable along the corresponding section of the gradient. Absolute curve height indicates the strength of each effect. (*a*) Functional β-diversity increased gradually with tree cover, especially above 25% tree cover, where the slope became steeper, indicating that dense hedgerows are not only highly dissimilar to herbaceous field margins, but also more dissimilar to each other. (*b*) Functional β-diversity was not affected by canopy height below 10 m but then increased very steeply with canopy height. (*c*) Field margins with very low values of herbicide spraying were highly dissimilar to each other, as indicated by the steep slope. Then, functional homogenization occurred with increasing herbicide spraying intensity, as indicated by the flattening of the curve around 2%, before β-diversity increased again for higher values of herbicide spraying intensity. (*d*) Cropping intensity had a weaker effect on functional β-diversity; field margins with intermediate values of cropping intensity are more dissimilar to each other than field margins at both ends of the gradient.

Generalized dissimilarity models (GDMs) were all significant but explained a relatively small fraction of β-diversity (less than 10%; table 1). Taxonomic and functional β-diversity were highly correlated (*r* = 0.66) but functional β-diversity was affected by a greater number of environmental variables (table 1). Thus, we focus on functional β-diversity hereafter. Regarding field margin structure, tree cover and to a lesser extent canopy height were the most important predictors of functional β-diversity. Functional β-diversity increased gradually with tree cover (figure 3*a*), especially above 25% tree cover, where the slope became steeper, indicating that dense hedgerows are not only highly dissimilar to herbaceous field margins, but also more dissimilar to each other. Functional β-diversity was not affected by canopy height below 10 m but then increased very steeply with canopy height (figure 3*b*). In addition to these results, we provide species accumulation curves showing that hedgerows have higher species γ-diversity (at landscape scale) than herbaceous field margins (electronic supplementary material, figure S8). Regarding long-term management, herbicide spraying in field margins and to a much lesser extent cropping intensity in adjacent habitats were the most important predictors of functional β-diversity (table 1). Functional β-diversity showed a three-step pattern in response to herbicide spraying intensity (figure 3*c*): (1) field margins with very low values of herbicide spraying were highly dissimilar to each other as indicated by the steep slope, (2) functional homogenization occurred with increasing herbicide spraying intensity, as indicated by the flattening of the curve around 2%, and (3) β-diversity increased again for higher values of herbicide spraying intensity. In addition, cropping intensity had a weaker effect on functional β-diversity; fields margins with intermediate values of cropping intensity are more dissimilar to each other than field margins at both ends of the gradient (figure 3*d*). Finally, geographical distance had no impact on functional β-diversity (table 1).

**Table 1.** Results of generalized dissimilarity models (GDMs) analysing the taxonomic and functional β-diversity of herbaceous plant communities in field margins as a function of environmental variables and geographical distance, after a backward selection procedure retaining only the most influential predictors. Variable importance is quantified as the percentage change in deviance explained between a model fitted with the variable permuted and un-permuted.

	taxonomic β-diversity	functional β-diversity
model *p*-value	0.000	0.000
deviance explained (%)	9.32	9.45
*variable importance* (%)		
tree cover	44.94	71.39
canopy height		18.09
herbicide	17.97	38.22
cropping		11.01
geographical distance	29.86	

### (c) Effects of field margin structure and long-term management on functional composition

**Figure 4.**
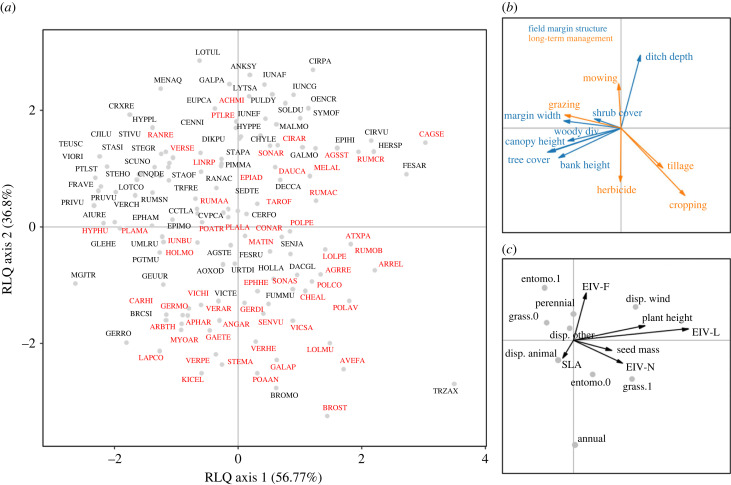
RLQ analysis performed on herbaceous plant communities of field margins. (*a*) Scores of species, (*b*) coefficients of environmental variables, and (*c*) traits on the first two axes. Red labels correspond to common weed species, based on [46]. Projection of non-weedy species versus common weeds on RLQ axes can be seen in electronic supplementary material, figure S10. Basic statistics for traits and environmental variables are given in electronic supplementary material, table S3. Species names are abbreviated using EPPO codes—see electronic supplementary material table S4 or EPPO Global Database (https://gd.eppo.int).

Monte-Carlo permutation tests revealed a significant link between traits and environment variables (*p*_max_ < 0.001). The first two axes of the RLQ analysis explained 94% of the total inertia (figure 4*a*). Basic statistics and coefficients of traits and environmental variables along RLQ axes are given in electronic supplementary material, table S3. The most influential variables on the first RLQ axis were tree cover, canopy height and cropping intensity in adjacent habitats (figure 4*b*). Herbaceous field margins or sparse hedgerows that are disturbed by high cropping intensity in adjacent habitats favoured grasses and plants with a high affinity for light and nutrients (figure 4*b,c*; electronic supplementary material, figure S9). Species associated with such field margins are mostly common weeds such as *Calystegia sepium* (CAGSE), *Cirsium vulgare* (CIRVU), *Rumex crispus* (RUMCR), *Arrhenatherum elatius* (ARREL), *Avena fatua* (AVEFA), *Anisantha sterilis* (BROST) and *Lolium multiflorum* (LOLMU) (figure 4*a*; electronic supplementary material, figure S10). On the other hand, denser and less-disturbed hedgerows favoured smaller, shade-tolerant, and non-weedy species (figure 4*b,c*; electronic supplementary material, figure S9) such as *Circaea lutetiana* (CJILU), *Teucrium scorodonia* (TEUSC), *Ajuga reptans* (AIURE), *Potentilla sterilis* (PTLST), *Fragaria vesca* (FRAVE), *Viola riviniana* (VIORI), *Stellaria holostea* (STEHO), *Primula vulgaris* (PRIVU), *Moehringia trinervia* (MGJTR) and *Polygonatum multiflorum* (PGTMU) (figure 4*a*, electronic supplementary material, figure S10).

The most influential variables on the second RLQ axis were ditch depth, mowing and herbicide spraying intensity (figure 4*b*). Field margins with deeper ditches, higher mowing intensity, and lower herbicide spraying intensity favoured hygrophilous, perennial and entomogamous species (figure 4*b,c*; electronic supplementary material, figure S9) such as *Lotus pedunculatus* (LOTUL), *Angelica sylvestris* (ANKSY), *Galium palustre* (GALPA), *Lythrum salicaria* (LYTSA), *Cirsium palustre* (CIRPA), *Oenanthe crocata* (OENCR), *Pulicaria dysenterica* (PULDY) and *Achillea millefolium* (ACHMI), but also rushes such as *Juncus acutiflorus* (IUNAF) and *Juncus conglomeratus* (IUNCG) (figure 4*a*). Conversely, field margins with no or shallow ditches, lower mowing intensity and higher herbicide spraying intensity favoured common weeds characterized by an annual life cycle and wind- or self-pollination modes (figure 4*b,c*; electronic supplementary material, figure S9), such as *Anisantha sterilis* (BROST), *Avena fatua* (AVEFA), *Bromus hordeaceus* (BROMO), *Poa annua* (POAAN), *Galium aparine* (GALAP), *Stellaria media* (STEMA), *Veronica persica* (VERPE), *Veronica hederifolia* (VERHE), *Kickxia elatine* (KICEL) and *Lapsana communis* (LAPCO) (figure 4*a*; electronic supplementary material, figure S10).

## 4. Discussion

### (a) How to promote local plant diversity in field margins?

Regarding field margin structure, tree cover was the major factor explaining both taxonomic and functional α-diversity and functional composition of plant communities in field margins. Field margins with greater tree cover were characterized by lower taxonomic and functional α-diversity. Similarly, Dainese *et al*. [47] found lower taxonomic α-diversity in hedgerows compared with herbaceous margins. Further, we found that denser hedgerows harboured mostly small and shade-tolerant species associated with woody habitats, and only a few common weeds, in line with previous studies [24,26,48]. Shady conditions prevented the growth of more common and light-demanding species but favoured rarer forest species in denser hedgerows, which explains their lower α-diversity. In addition, plant communities of denser hedgerows were probably the most severely affected by agricultural intensification that occurred in the study area, leading to higher extinction and fewer colonization events of forest species [18]. Indeed, forest species are very sensitive to agrochemical drifts [49,50]. Forest species probably also suffer from microclimate changes and reduced connectivity between hedgerows and source habitats (forests and woodlots), caused by habitat destruction and increased arable field size [50,51]. Promoting or restoring plant α-diversity in hedgerows requires preserving woodland, hedgerows and their connectivity in agricultural landscapes [50,52].

Beyond tree cover, ditch depth was another major driver of taxonomic and functional α-diversity and functional composition of field margins. Field margins with deeper ditches harboured more diverse plant communities including many non-weedy and perennial species, in line with previous studies [24,25]. In our case, this is most likely the result of several factors. First, ditches provide unique habitats characterized by slopes and high soil moisture, and we logically found that hygrophilous species were strongly associated with deep ditches. Second, ditch depth is also related to a specific set of management practices. Field margins with deep ditches are frequently mown, and a prefectural decree prohibits the use of herbicides near ditches in the study area since 2005. These management practices could explain why insect-pollinated perennials were associated with deep ditches. Mowing may favour perennial species with subterranean or near-surface buds that can regrow afterwards [53]. Mowing can also promote insect-pollinated plants (especially in eutrophic conditions) by reducing the dominance of competitive grasses [54,55]. In addition, herbicide treatments select annual species that can escape disturbances owing to their short life cycles, especially those germinating and flowering all year round [56,57]. Physiological tolerance to herbicide treatments is also increasingly common among weed species [58].

Regarding long-term management, grazing was the most important driver of taxonomic and functional α-diversity, alongside mowing. Field margins with higher grazing intensity over time supported more diverse plant communities, again likely the result of several factors. First, direct effect of grazing on plant communities can prevent the dominance of competitive species and reduce soil fertility [59]. In line with this interpretation, we found that small plants producing small seeds (i.e. less competitive), as well as less nitrophilous species, were associated with higher grazing intensity. Second, higher grazing intensity implies higher recurrence of temporary grasslands in the crop rotation of adjacent fields. Thus, field margins with higher grazing intensity are probably less disturbed by agrochemical drifts in the long term, further favouring less nitrophilous species. Plant spillover from grasslands could also increase plant diversity in field margins (e.g. [10]), although temporary grasslands are generally species-poor in our study area. Whether the positive effect of grazing is direct or indirect, we point out that too much grazing pressure could have adverse effects on both herbaceous and shrub layers.

Surprisingly, margin width did not affect taxonomic and functional α-diversity of plant communities in field margins, probably because the range is not wide enough (i.e. less than 4 m). Nonetheless, the beneficial effects of increasing margin width should not be overlooked (e.g. [24,25]). In addition, we did not detect a negative effect of herbicide spraying on α-diversity, in contrast with other studies [9,60,61]. Herbicide spraying was detrimental to perennial and non-weedy species. On the other hand, many weed species were probably able to escape herbicide treatments owing to their short life cycle and phenological plasticity, and may also benefit from reduced competition with perennial and non-weedy species.

### (b) How to prevent biotic homogenization of field margins at landscape scale?

Our study shows that both field margin structure and long-term management practices affect taxonomic and functional β-diversity of herbaceous plant communities in field margins. Regarding field margin structure, tree cover was the most important driver of taxonomic and functional β-diversity. Notably, field margins with lower α-diversity were those contributing most to β- and γ-diversity. Denser hedgerows were locally less diverse than herbaceous field margins or sparser hedgerows, but collectively more dissimilar to each other, which is explained by the higher rarity of forest species. To our knowledge, most studies have focused on α-diversity but very few have assessed the contribution of different habitat types to β- or γ-diversity in agricultural landscapes. Boutin *et al*. [62] showed that natural hedgerows had higher γ-diversity and harboured more species of conservation value than recently planted hedgerows. Walker *et al*. [63] also found that green lanes (i.e. two parallel hedgerows separated by a central track) had higher γ-diversity than single hedgerows, and favoured more shade-tolerant and hygrophilous species. Conservation efforts should pay particular attention to denser and older hedgerows, as they harbour more unique sets of species or life strategies and contribute most to increasing β- and γ-diversity of field margins (at landscape scale). In addition, our results show that it is important to preserve a gradient of tree cover (beyond 25%) and canopy height (beyond 10 m) among hedgerows in the landscape. Indeed, the effects of tree cover and canopy height on functional β-diversity were almost linear beyond these thresholds, which indicates that each field margin in these portions of environmental gradients is in some ways unique and contributes to β-diversity.

Regarding long-term management over 20 years, herbicide spraying was the most important driver of taxonomic and functional β-diversity, despite the absence of effect on α-diversity. Herbicide spraying affected β-diversity in two ways. First, at intermediate values of herbicide spraying, we observed a decline in β-diversity associated with a shift from non-weedy species to common weeds—mostly annual species that do not depend on insects for pollination, such as *Galium aparine*, *Anisantha sterilis* and *Avena fatua*. This is not surprising as common weeds are best at escaping herbicide treatment and developing physiological tolerance to herbicides [58]. At one end of the gradient, unsprayed margins harbour dissimilar sets of species, mostly non-weedy, perennial and insect-pollinated forbs providing more stable trophic and habitat resources for animals (e.g. [64,65]). These results strongly suggest that long-term herbicide spraying undermines both ecological and agronomic functions of field margins, in line with the findings of Smith *et al*. [9]. At the other end of the gradient, among the resulting species pool dominated by common weeds, varying herbicide spraying intensities (from intermediate to high values) maintained higher β-diversity. This can be explained by the diversity of life strategies in common weeds. Common weed species are distributed along the gradient of herbicide spraying intensity based on their level of physiological tolerance or their ability to escape increasingly frequent treatments.

Cropping intensity in adjacent habitats had a relatively smaller effect on taxonomic and functional β-diversity of herbaceous plant communities. Cropping intensity favoured common weed species as well, mainly annual grasses with a strong affinity for nutrients. This is probably due to increased chemical fertilizer drifts, soil eutrophication and acidification in field margins [66], and weed spillover from arable fields. Possibly, the dominance of grasses in field margins and the intense weed control in conventional fields lead to a depletion of pollinators in field margins and their vicinity, further disadvantaging insect-pollinated forbs [11,25]. All in all, field margins should be protected from both herbicides and chemical fertilizers. This can be achieved by widening field margins, by establishing buffer strips at the interface between arable fields and margins (as recommended for watercourses), and/or by reducing agrochemical inputs to arable fields (e.g. organic farming).

In conclusion, we could not confirm our hypothesis that selection for weedy species in structurally simple and most disturbed field margins leads to both local impoverishment (i.e. decrease in α-diversity) and biotic homogenization at landscape scale (i.e. decrease in β-diversity). It appears that a diversity of weed species and life strategies in such field margins helped maintain α- and β-diversity, despite the removal of rarer and more disturbance-sensitive species. However, this cannot be regarded as a positive result for biodiversity conservation and ecosystem functioning. Conservation of arable plants should take place in arable fields, whereas field margins are one of the few habitats that can be devoted to the conservation of rarer and more disturbance-sensitive species and their associated ecological functions. We also emphasize that we surveyed plant communities in 2015, which are most likely a subset of plant communities that were present before agricultural intensification began in the 1950s. Many local extinctions probably have already occurred, leaving mostly those species best able to cope with strong anthropogenic disturbances. In any contemporary study, we most likely underestimate the negative impacts of agricultural intensification on α*-* and β-diversities, especially considering the existence of shifting baseline syndrome [67]. For instance, Kempel *et al*. [68] revealed thousands of local extinctions of rare and threatened species between 1960 and 2001 across many habitats, including hedgerows and herbaceous field margins. Once the damage is done, we are no longer able to accurately estimate (or even detect) the impact [69], unless historical reference data are available.

### (c) A multi-faceted approach for more robust conservation and restoration decisions

Our study revealed significant discrepancies between the drivers of α*-* and β-diversities of plant communities in field margins, highlighting that both facets should be considered when making conservation and restoration decisions. In addition, we did not find many forms of antagonisms or synergies between the preservation of α- versus β-diversity. Most drivers affecting α-diversity did not affect β-diversity, and *vice versa*. This implies that it is possible to promote both α- and β-diversity by adopting different sets of management practices. Strikingly, by focusing solely on α-diversity, one could conclude that plant communities in dense hedgerows are poorer than those in herbaceous margins, and that herbicide spraying and agrochemical drifts do not affect plant diversity. The study of β-diversity painted a very different picture, as it revealed that dense hedgerows strongly contribute to the preservation of plant diversity at landscape scale, and that herbicide spraying and agrochemical drifts potentially lead to biotic homogenization of plant communities in field margins.

By contrast to α- versus β-diversity, taxonomic and functional diversities yielded the same conclusions for biodiversity conservation. Both indices were strongly and positively correlated, indicating low functional redundancy between plant species. Nonetheless, many studies revealed that this is not always the case. For instance, taxonomic diversity can increase more than functional diversity, if additional species are functionally redundant (e.g. [70,71]). Similarly, functional diversity can decrease more than taxonomic diversity, if functionally redundant species are less affected than (or even replacing) more unique species (e.g. [72]). On the other hand, contrary to the limited benefit of measuring functional diversity in our case, the study of functional *composition* did provide major and complementary information for both biodiversity conservation and agricultural production. Notably, it revealed that the removal of hedgerows, long-term herbicide spraying and cropping intensity in adjacent habitats select for competitive and weedy species (characterized by higher stature, stronger affinity for light and nutrients, and annual life cycle) in field margins. This major shift in community composition, with strong implications for both biodiversity conservation and ecosystem functioning, cannot be detected with α- and β-diversity indices.

### (d) A word of caution

Although long-term survey of communities is essential, the absence of a decline in α- or β-diversity over time does not reliably rule out ongoing biodiversity loss. Indeed, Alignier [19] showed that the overall β-diversity of plant communities in surveyed field margins did not decline between 1994 and 2015. Revisiting the same dataset, but this time assessing the drivers of β-diversity and functional composition, we showed that some field margin structures and long-term management practices are maintaining plant β-diversity and species of higher conservation value, whereas others are potentially leading to biotic homogenization by selecting for common weeds. Such information is essential for making better conservation and restoration decisions before it is too late.

## Data Availability

Data are available via the Dryad Digital Repository (https://doi.org/10.5061/dryad.79cnp5hz9) [73]. The data are also provided in electronic supplementary material [74].
